# Acute gastric obstruction following cardiopulmonary bypass in a patient with an adjustable gastric band

**DOI:** 10.1186/1749-8090-10-S1-A170

**Published:** 2015-12-16

**Authors:** Raed Azzam, Abdelrahman Abdelbar, Andrew Brazier, Bilal H Kirmani, Ragheb Hasan

**Affiliations:** 1 Department of Cardiothoracic Surgery, Manchester Heart Centre, Manchester Royal Infirmary, Oxford Road, Manchester, M13 9WL, UK

## Background/Introduction

Obesity is a major problem with the number of obese patients presenting to cardiac surgery is ever increasing. Laparoscopic Adjustable Gastric Banding (LAGB) is one modality commonly used to mechanically reduce food intake and also promotes early satiety. Late complications of the device may occur in up to 20% of cases; increasing up to 30% in pregnancy. We report the first case of a late complication of LAGB after cardiopulmonary bypass.

## Aims/Objectives

A 51 year old female with symptomatic, severe mitral valve regurgitation presented for elective mitral surgery. She had a history of atrial fibrillation, and insertion of LAGB two years previously. The patient underwent successful mitral valve repair using a 30 mm Cosgrove ring. Trans-oesophageal echocardiogram showed a competent valve, although transgastric views were avoided to prevent displacement of the gastric band. Post-operative recovery was uneventful and the patient was stepped down to the ward promptly. Diuretics were discontinued when the patient returned to pre-operative weight.

## Method

The following day the patient complained of nausea and non-bilious vomiting. Her symptoms persisted despite cessation of opioid analgesia and commencing antiemetics. There were no significant clinical findings. Blood biochemistry and plain x-ray films were unremarkable.

## Results

A gastrografin swallow showed that contrast failed to pass through the gastro-oesophageal junction. The gastric band balloon was deflated by 0.6 ml. Oral intake was resumed and she was discharged home. There was no residual dysphagia at follow up.

## Discussion/Conclusion

The most common causes of postoperative nausea and vomiting are drug-induced emesis, paralytic ileus, obstruction or mesenteric ischaemia. Where patients have been stable with an LAGB in situ for some time, this can make the diagnosis of gastric inflow obstruction challenging.

One possible explanation for the acute deterioration of an established LAGB following cardiac surgery is subacute oedema in the stomach wall at the site of the gastric band. Fluid retention and post-operative gut distension. Gastric band obstruction should be considered if such patients develop upper gastro-intestinal. An early gastrografin swallow should be considered.

## Consent

Written informed consent was obtained from the patient for publication of this abstract and any accompanying images. A copy of the written consent is available for review by the Editor of this journal.

